# A comparative study to assess the effect of amikacin sulfate bladder wash on catheter-associated urinary tract infection in neurosurgical patients

**DOI:** 10.4103/0972-5229.53110

**Published:** 2009

**Authors:** Sumi Zacharias, Srinivas Dwarakanath, Meena Agarwal, Bhavani Shankar Sharma

**Affiliations:** **From:** College of Nursing, New Delhi, India; 1 Department of Neurosurgery, All India Institute of Medical Sciences, New Delhi, India

**Keywords:** Amikacin bladder irrigation, bladder wash, catherer-associated urinary tract infection

## Abstract

**Background:**

The indwelling urinary catheter is an essential part of modern medical care. Unfortunately, when poorly managed, the indwelling catheter may present a hazard to the very patients it is designed to protect. Catheter-associated urinary tract infection (CAUTI) is the most common nosocomial infection in hospitals and nursing homes.

**Aims and Objectives:**

The primary objective was to study the effect of amikacin sulfate bladder wash on CAUTI in neurosurgical patients. The other objectives were to study the various organisms causing CAUTI and their antibiotic sensitivity and resistance pattern.

**Materials and Methods:**

This was a prospective randomized controlled study performed on 60 patients who met the inclusion criteria at the neurosurgical intensive care of the All India Institute of Medical Sciences between June and December 2006. The patients were randomized into two groups – one was the trial group which received amikacin bladder wash, while the other was the control group that did not receive any bladder wash.

**Results:**

Forty percent of the subjects in the control group developed CAUTI, while none of the subjects in study group developed CAUTI. (Fisher's exact test, *P* value < 0.001) *Pseudomonas aeruginosa* (51%) was the commonest pathogen.

**Conclusions:**

Amikacin sulfate bladder wash was effective in preventing CAUTI. It can thus decrease the antibiotic usage thereby preventing the emergence of antibiotic resistance.

## Introduction

The indwelling urinary catheter is an essential part of modern medical care and a variety of different indwelling urinary catheters can be used for various purposes. Each year, urinary catheters are inserted in more than five million patients in acute-care hospitals and extended-care facilities.[1]

Unfortunately, when poorly managed, the indwelling catheter may present a hazard to the very patients it is designed to protect. Catheter-associated urinary tract infection (CAUTI) is the most common nosocomial infection in hospitals and nursing homes, comprising >40% of all institutionally acquired infections.[2–6] Interventions such as topical meatal antimicrobials, disinfectants added to the urinary drainage bag, and antimicrobials coatings for catheters have not been shown to decrease the incidence of UTI.[7] An effective measure to prevent the CAUTI has not yet been developed. The most common organisms responsible for CAUTI are *Escherichia coli*, *Candida* spp., *Klebsiella pneumoniae*, *Streptococcus agalactiae*, *Enterococcus faecalis*, and *Pseudomonas aeruginosa*. Amikacin has proven to be the most effective antibiotic in preventing the growth of all these species.[8]

Previous studies have shown that using individualized regimens for bladder washouts minimizes the infection rate and catheter blockage, thus reducing the need for frequent recatheterization.[9] Since there is no significant systemic absorption of antibiotics used for bladder irrigation,[10] prophylactic bladder irrigations are considered to be safe. We performed an extensive review of literature which did not reveal any study done on the effectiveness of amikacin sulfate bladder wash.

## Materials and Methods

This study was performed on a total of 60 catheterized patients who were admitted to the Neurosurgery Department of AIIMS, New Delhi, during June–December 2006 and met the inclusion criteria [Appendix 1]. The subject data sheet and procedure of amikacin sulfate bladder wash was developed under the guidance of a guide (Author 3) and co-guides (Authors 2 and 4), and was further validated by six experts from the Departments of Neurosurgery, Microbiology, and Nursing, AIIMS.

Informed written consent was taken from the patients or patients' relatives after providing appropriate information to the concerned. Confidentiality of the data was ensured. They were randomized after catheterization to either of the two groups – study and the control groups. Then urine samples were sent within 24 hours for culture and sensitivity (C/S), in case of positive C/S, patients were excluded from the study [Appendix 2]. Study group received amikacin sulfate bladder wash twice daily under strict aseptic precautions and the control group did not receive bladder wash [Appendix 3]. Urine C/S was performed on days 3, 7, and then weekly till the removal of catheter or discharge. Both groups received standard catheter care including perineal care; the only difference was the bladder wash. The researcher (Author 1) performed the bladder wash on all patients to eliminate bias. CAUTI was diagnosed when it met the respective criteria [Appendix 4].

### Statistical methods

Descriptive and inferential statistical methods were used. Data were analyzed using SPSS-10^th^ version. A probability of <0.05 was accepted as significant. For continuous variables having normal distribution, data were summarized using mean±SD, and the groups were compared using independent *t* test. Range and median were used for all continuous variables having non-normal distribution (age and duration of catheterization) and the two groups were compared using Mann-Whitney U test. Frequency and percentage were used for all categorical variables and the groups were compared using Pearson's chi-square test and Fisher's exact test.

The study was conducted following the approval of ethics committee of All India Institute of Medical Sciences (AIIMS).

## Results

### Demographic profile of patients

Age of the study subjects ranged from 18–68 years [Table 1]. The groups were homogenous in terms of age, sex, level of consciousness, size of catheter and duration of catheterization, and systemic usage of steroids and antibiotics. Bladder wash was well tolerated by all the subjects in the study group.

**Table 1:** Age, sex, and duration of catheterization (n=60)

Variables	Study group *n* = 30	Control group *n* = 30	*P* value
Age (years)	19–65 (38)	18–68 (42.5)	0.399
Males (%)	16 (53)	16 (53)	1.0
Females (%)	14 (47)	14 (47)	-
Duration of catheterization (days)	3–29 (7)	4–20 (8)	0.823

### Incidence of catheter-associated urinary tract infection and predisposing factors

**Figure 1:**
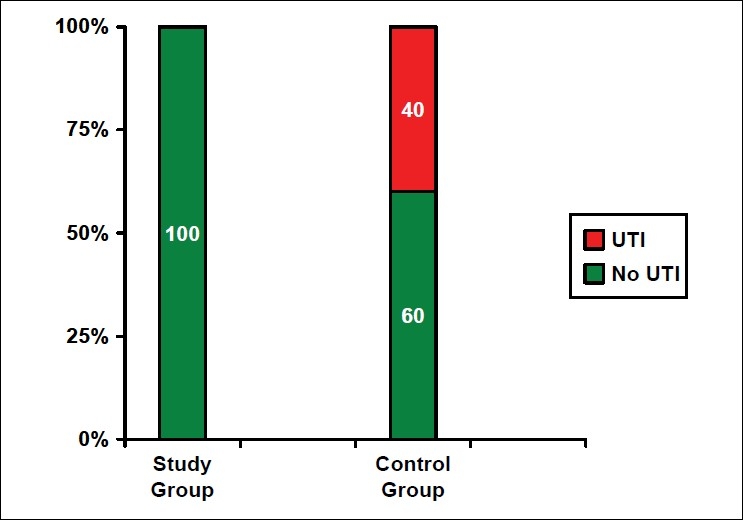
Effect of amikacin sulfate bladder wash on catheter-associated urinary tract infection

Incidence of CAUTI was 40% in the control group [Figure 1]. None of the subjects from the bladder wash group developed CAUTI. Amikacin bladder wash was effective in preventing CAUTI (*P* < 0.001). Lower levels of consciousness (i.e. the Glasgow coma scale (GCS)) increased the risk of developing CAUTI (*P* = 0.026) [Table 2].

**Table 2:** Risk factors for catheter-associated urinary tract infection (n=60)

Factors	UTI-positive group n = 12	UTI-negative group n = 18	*P* value
Age (years)	18–68 (46.5)	20–55 (41.5)	0.280
Sex			0.457
Females	7 (50)	7 (50)	
Males	5 (31.3)	11 (68.7)	
Motor score of GCS			0.026^*^
M 1–3	5 (83.4)	1 (16.6)	
M 4–6	7 (29.2)	17 (70.8)	
Catheter size			0.131
12	0	2 (100)	
14	7 (36.8)	12 (63.2)	
16	5 (71.4)	2 (28.6)	
18	0	2 (100)	
Duration of catheterization	6–20 (9)	4–14 (7.5)	0.135
Systemic antibiotics			0.255
Yes	12 (44.4)	15 (55.6)	
No	0	3 (100)	
Steroids			0.643
Yes	7 (36.8)	12 (63.2)	
No	5 (45.5)	6 (54.5)	

### Organism and sensitivity profile

**Figure 2:**
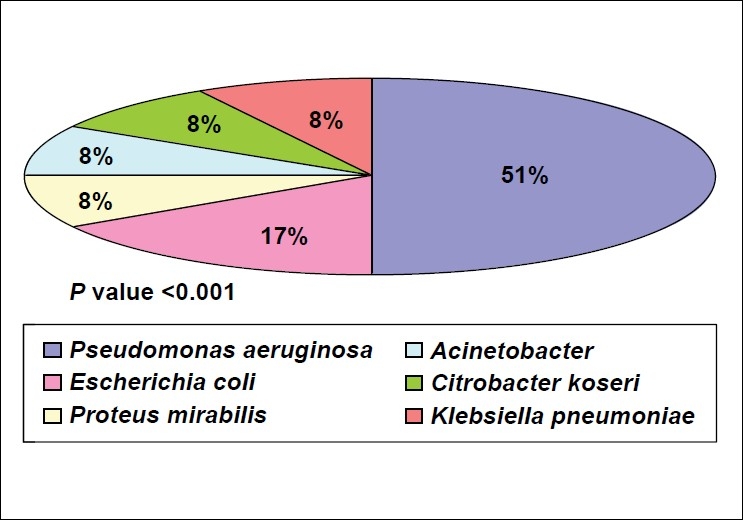
Pathogens causing catheter-associated urinary tract infection

*P. aeruginosa* was the most common organism responsible for 51% of CAUTI [Figure 2]. *P. aeruginosa* was completely sensitive to amikacin sulfate, cefaperazone plus sulbactam, and piperacillin plus tazobactam, while it was completely resistant towards ceftazidime.

## Discussion

The present study is the first of its kind in which the effect of amikacin bladder wash has been analyzed. Efficiency of bladder irrigation using various different solutions have been studied in the past in an attempt to reduce the incidence of CAUTI, majority of the investigators found it to be a time consuming and costly procedure that did not have an impact on CAUTI.[11–15] In contrast, the present study revealed that amikacin bladder wash is effective in preventing CAUTI. In previous studies, the incidence of CAUTI ranged from 11.0–73.3%.[16,17] In the present study, incidence of CAUTI was 40% since those who were catheterized for less than three days were excluded in the study. This inclusion criterion might be the chief reason for a higher incidence of CAUTI in the present study.

CAUTI increases the burden of the patient in terms of increased morbidity and mortality, prolonged hospital stay, and cost of the tests and medicines.[17–19] Tambyah *et al.*,[20] found that CAUTI had been responsible for an additional of USD589 per CAUTI in diagnostic tests and in medications. The present study reveals that amikacin sulfate bladder wash is effective in preventing CAUTI. As a vial of amikacin sulfate (500 mg) costs INR58 (approximately USD1.4), this is very cost effective especially in a developing country like ours.

Puri *et al.*,[21] in their study said that the risk was significantly higher for females, elderly patients, critically ill patients, and those on prolonged catheterization. The present study showed only severity of the disease (low motor score of GCS) as a statistically significant risk factor. This might be since a lower GCS corresponds to the severity of tissue injury, where there is hypermetabolism and increased protein catabolism, which eventually leads to decreased immunity that makes the person more susceptible to infections. However, the present study did not show any influence of sex, age, catheter size, duration of catheterization, and systemic use of antibiotics and steroids. This might be because of the small sample size and inclusion of long-term catheterized patients only.

Pathogenic organisms responsible for CAUTI and their antibiotic sensitivity pattern vary with time. In a study, Jha *et al.*,[22] found that most common organisms responsible for CAUTI were *E. coli* (49%), *S. aureus* (23%), Proteus spp. (3.6%), *Klebsiella* (9.71%), *Pseudomonas* (0.8%), and *Citrobacter* (2.8%). Whereas in the present study, *Pseudomonas* (51%), *E. coli* (17%), *Proteus* spp., *Citrobacter*, *Klebsiella*, and *Acinetobacter* (8% each) were the most common. Similarly, antibiotic resistance pattern also varied. In their study, Taneja *et al.*,[23] found the highest frequency of antibiotic resistance was for ciprofloxacin (68.6%) followed by netilmicin (60.7%), ceftazidime (58.8%), imipenem (43.7%), amikacin (43.1%), and piperacillin (39.2%). In the present study, the pattern of antibiotic resistance was ceftazidime (100%), netilmicin (83%), imipenem (75%), ciprofloxacin (75%), and meropenem (60%).

In this study, we have shown that amikacin sulfate bladder wash is very effective in preventing CAUTI. Thus it can be included in the routine catheter care, especially if catheterization is needed for more than five days. It is easy to implement and cost effective. The main limitation of this study was the small sample size and the fact that the main researcher was not blinded to the study.

## Conclusions

There is a varying pattern of antibiotic sensitivity and resistance in different institutions. The most important thing to note is the fact that the bacteria have started developing resistance to higher antibiotics. This is very important since the indiscriminate use of antibiotics can lead to resistance, thus potentially endangering the life of a patient. This increasing resistance calls for immediate measures to use methods other than oral or parenteral antibiotics in CAUTI. We believe that this study is important because if used can decrease the development of CAUTI.

## Appendix 1

**Inclusion criteria**

- Informed consent
- Age above 18 years
- Patients available within 24 hours of catheterization

## Appendix 2

**Exclusion criteria**

- Past history of recurrent UTI
- Urine culture positive within 24 hours after enrolment in to the study
- Immunocompromized patients (AIDS, chemotherapy)
- History of diabetes
- Patients with renal insufficiency
- Catheter removed before third day

## Appendix 3

**Amikacin sulfate bladder wash**

Hundred milligrams amikacin sulfate is added to 500 ml normal saline and 250 ml of this solution is instilled into the bladder through a Foleys catheter (with drainage tube clamped) using an IV set. Solution is allowed to remain in the bladder for 15 minutes and then drained. This is done twice daily.

## Appendix 4

**Catheter-associated urinary tract infection–**

The diagnosis of catheter-associated urinary tract infection can be made when the urine culture shows 10^5^ CFU/ml of urine from a catheterized patient.
